# What Are the Top 10 Research Questions in the Treatment of Inflammatory Bowel Disease? A Priority Setting Partnership with the James Lind Alliance

**DOI:** 10.1093/ecco-jcc/jjw144

**Published:** 2016-08-09

**Authors:** Ailsa L. Hart, Miranda Lomer, Azmina Verjee, Karen Kemp, Omar Faiz, Ann Daly, Julie Solomon, John McLaughlin

**Affiliations:** a IBD Unit, St Mark’s Hospital, Harrow, Middlesex, UK; b Diabetes & Nutritional Sciences Division, Kings College London, London, UK; c Bowel Disease Research Foundation, Royal College of Surgeons of England, London, UK; d Department of Gastroenterology, Manchester Royal Infirmary, Central Manchester University Hospitals NHS Trust, Manchester, UK; e Birmingham Women's Hospital, Birmingham Women’s NHS Foundation Trust, Birmingham, UK; f British Society of Gastroenterology, London, UK; g University of Manchester Institute of Inflammation and Repair, Salford, UK

**Keywords:** Research priorities, inflammatory bowel disease, James Lind Alliance

## Abstract

**Background and Aims:**

Many uncertainties remain regarding optimal therapies and strategies for the treatment of inflammatory bowel disease. Setting research priorities addressing therapies requires a partnership between health care professionals, patients and organisations supporting patients. We aimed to use the structure of the James Lind Alliance Priority Setting Partnership, which has been used in other disease areas, to identify and prioritise unanswered questions about treatments for inflammatory bowel disease.

**Methods:**

The James Lind Priority Setting Partnership uses methods agreed and adopted in other disease areas to work with patients and clinicians: to identify uncertainties about treatments; to agree by consensus a prioritised list of uncertainties for research; then to translate these uncertainties into research questions which are amenable to hypothesis testing; and finally to take results to research commissioning bodies to be considered for funding.

**Results:**

A total of 1636 uncertainties were collected in the initial survey from 531 respondents, which included 22% health care professionals and 78% patients and carers. Using the rigorously applied processes of the priority setting partnership, this list was distilled down to the top 10 research priorities for inflammatory bowel disease. The top priorities were: identifying treatment strategies to optimise efficacy, safety and cost-effectiveness; and stratifying patients with regard to their disease course and treatment response. Diet and symptom control [pain, incontinence and fatigue] were also topics which were prioritised.

**Conclusions:**

A partnership involving multidisciplinary clinicians, patients and organisations supporting patients has identified the top 10 research priorities in the treatment of patients with inflammatory bowel disease.

## 1. Introduction

The inflammatory bowel diseases [IBD], which include Crohn’s disease and ulcerative colitis, are chronic relapsing and remitting diseases that cause inflammation of the gastrointestinal tract. The aetiology of IBD is unknown, but in genetically predisposed hosts there appears to be an intestinal mucosal immune reaction to an environmental factor, with the gut microbiota playing a central role.^1^ These diseases are increasing in incidence and carry a burden of symptoms including abdominal pain, diarrhoea and sometimes faecal incontinence, fatigue, extra-intestinal symptoms including joint pains, skin lesions and eye symptoms, and an increased risk of cancer. Treatment of IBD involves a multidisciplinary approach to management, with medical therapies and surgery playing important roles. All therapies are underscored by supporting the psychological needs of the patient.

Many basic questions and uncertainties remain regarding the cause of these inflammatory bowel conditions and the optimal therapies and strategies for treatment. Research to advance the management of these diseases requires resources. However, it is difficult to prioritise research proposals and allocate resources, because of the large number of competing ideas, difficulty in measuring the impact of research and inherent uncertainty about the outcome of research. What are the research priorities for these complex diseases? And how do research-funding bodies decide the research priorities in any particular disease area? Research priorities are often set by academics or by the pharmaceutical industry; however, are these groups best placed to ensure that resources for research are focused on the clinically-relevant and meaningful issues for clinicians and patients?

In a recent survey of allocation of funds by medical research funding bodies in the UK, most [31 out of 52] rely on researchers submitting their research ideas, which are then peer-reviewed, before decisions are made on funding allocations.^2^ However in complex diseases requiring multidisciplinary input, such as IBD, it may be that key questions asked by different members of the IBD team [gastroenterologists, colorectal surgeons, IBD specialist nurses and dietitians] are overlooked. In particular, the patients’ perspective on research priorities is rarely taken into account and this should be central to decisionmaking and setting priorities for research.

An initiative in the UK which supports a partnership between health care professionals, patients and organisations supporting patients in setting research priorities is the James Lind Alliance. The James Lind Alliance was set up in 2004 and is managed by the National Institute of Health Research Evaluation, Trials and Studies Coordinating Centre in the UK.^3^ It has the goal of bringing together clinicians, patients and other stakeholders to set research priorities. It focuses on questions related to treatments or treatment pathways for a given disease; aetiology and underpinning science are not in scope. It aims to identify unanswered questions related to treatment within a particular specialty, and then ranks these ‘treatment uncertainties’ in order of priority, with the final output being a ‘top 10’ of research priorities for that given disease area. ‘Treatment uncertainties’ are defined either when up-to-date systematic reviews of treatments or treatment strategies demonstrate that uncertainty exists, or when there are no up-to-date systematic reviews addressing uncertainty of treatments or treatment strategies. The advantage of the James Lind Alliance is that it provides a framework with a transparent, democratic and reproducible process, and a panel of advisers who guide the process. It is recognised as the gold standard in setting research priorities. The pharmaceutical industry and non-clinical academics are not involved in the process, to ensure that identified priorities are those that matter most to patients and their clinicians.

This has successfully worked in other disease areas [over 30 diseases areas have been covered] to identify and prioritise treatment uncertainties that are important to patients and clinicians. For example, in other chronic diseases such as diabetes, asthma and Parkinson’s disease,^4–6^ the James Lind Alliance has produced comprehensive lists of the top 10 research priorities as devised by clinicians, patients and their carers. All of the unanswered questions discovered by this process are important, regardless of their final position in the list of priorities. The agreement of a list of research priorities marks the beginning of the next stage of work which is to promote the priorities to key groups such as research funders, researchers, patients and carers and the wider research community. Several of the uncertainties identified and prioritised in the work by the James Lind Alliance have been considered by funding bodies and have or are likely to be funded as research projects in the near future. No previous Priority Setting Partnership had addressed any aspect of gastrointestinal disease.

The aim of this study was to devise a list of the key research priorities regarding treatment of IBD, as seen by clinicians, patients and their support groups, using a structure established by the James Lind Alliance.

## 2. Methods

**Figure 1.**
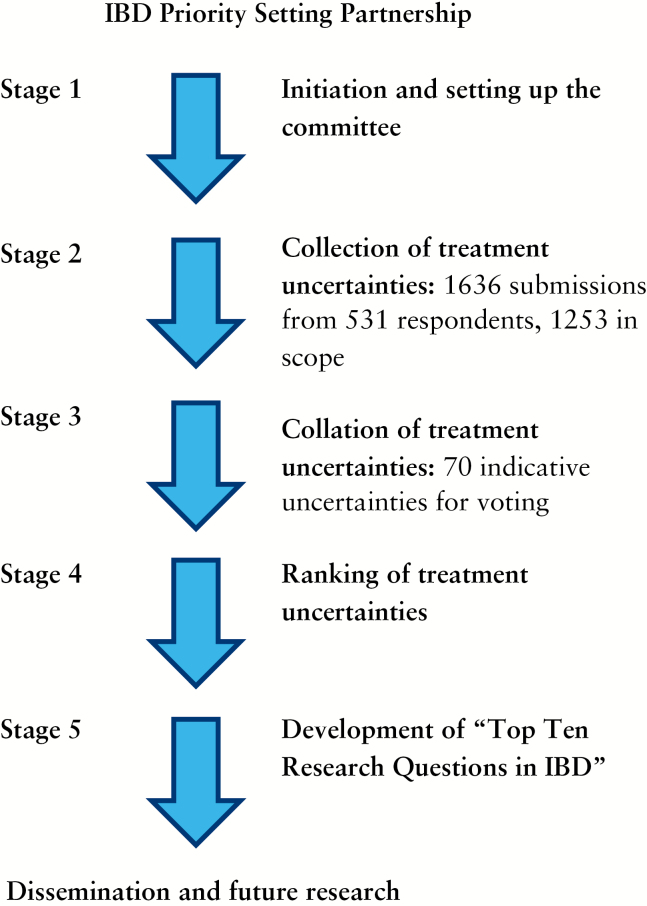
Outline of James Lind Alliance Process.

An outline of the James Lind Alliance process is detailed at [http://www.jla.nihr.ac.uk/guidebook] and shown in Figure 1. Details of the methods agreed and adopted can be found in a number of publications^7^^,^^8^ and therefore only a brief summary is presented here. The objectives of the IBD priority-setting partnership are: to work with IBD patients and clinicians to identify uncertainties about IBD treatments; to agree by consensus a prioritised list of those uncertainties for research; then to translate these prioritised uncertainties into research questions which are amenable to hypothesis testing; to publish the results of the process; and finally to take the results to research commissioning bodies to be considered for funding.

### 2.1. Stage 1. Initiation and setting up the committee

From the outset it was recognised that many health care professionals are involved in the care of patients with IBD, including gastroenterologists, colorectal surgeons, IBD specialist nurses, dietitians and psychologists. The organisations and groups approached at the inception stage, and who agreed to participate, included the British Society of Gastroenterology [BSG], Association of Coloproctology of Great Britain [ACPGBI], Royal College of Nursing [RCN], British Dietetic Association [BDA], British Society of Paediatric Gastroenterology Hepatology and Nutrition [BSPGHAN], the UK inflammatory bowel disease charity organisation called Crohn’s and Colitis UK, the Crohn’s in Childhood Research Association [CICRA], the Primary Care Society for Gastroenterology, the charity committed to fighting all diseases that affect the gastrointestinal tract [CORE], the National Institute for Health Research Gastroenterology Speciality Group [NIHR GI SG], the National Institute for Health Research Children Speciality Group [NIHR CSG] and the James Lind Alliance [JLA]. A steering committee was established following an initial explanatory meeting and included two patients with IBD, two gastroenterologists, two inflammatory bowel disease specialist nurses, two colorectal surgeons, two dietitians, a representative from the UK inflammatory bowel disease charity organisation Crohn’s and Colitis UK, a representative of the James Lind Alliance and an administrator. The steering group defined the scope of the partnership and developed the protocol detailing the methods to be used based on established James Lind methodologies and adapted to IBD. The James Lind Alliance and the steering group assessed organisations for any potential conflict of interest that might have led to unacceptable bias if they were to participate, in keeping with the ethos of the James Lind Alliance. The British Society of Gastroenterology and Crohn’s and Colitis UK provided core funding for the project in equal shares, with other members of the committee contributing time and expertise or facilities at no cost.

### 2.2. Stage 2. Collection of treatment uncertainties

The aim of this stage was to collect treatment uncertainties [defined as ‘known unknowns’] by developing an initial survey questionnaire, in both electronic and paper formats. Participants were invited to submit up to five treatment uncertainties. The survey questionnaire was designed to capture uncertainty about treatments for IBD from patients, carers, IBD specialist nurses, gastroenterologists, surgeons and dietitians. The anonymous survey was developed and opened out between March and May 2014. The text within the survey was checked for ease of understanding by lay members of the steering group. Broad treatment categories were provided in order to guide participants and to make collation of submissions easier. A 2-week pilot of the survey was conducted by the IBD specialist nurse network and their patient panels. Completion of the survey was considered to imply consent to participate in the prioritisation process. Any uncertainties registered on the UK Database of Uncertainties about the Effects of Treatments [http://www.library.nhs.uk/duets/], Research Recommendations from Cochrane Systematic Reviews and the National Institute for Health and Care Excellence, but not represented in the list of uncertainties generated through the survey, were added to the list of uncertainties. The survey was advertised through a combination of direct emails and newsletters to members of the partner organisations, including Crohn’s and Colitis UK, and through links on relevant websites.

### 2.3. Stage 3. Collation of treatment uncertainties

The aim of this stage was to review the treatment uncertainties gathered in the collection stage and produce a ‘long list’ of treatment uncertainties for IBD. A professional and independent information analyst with previous experience of priority-setting partnerships was contracted to undertake this process. First, any non-questions [e.g. statements or comments] were removed, as were uncertainties not relating to IBD and uncertainties not related to treatment of IBD [e.g. aetiology, which is outside the James Lind scope]. Remaining uncertainties were reviewed and, where appropriate, any similar uncertainties were combined to create ‘indicative uncertainties’, which were refined into a standard format. Any indicative treatment uncertainties mentioned by only one respondent were removed. The full list and wording of the indicative uncertainties were then checked by members of the steering group and consensus was sought from members of the steering group if differences in opinion were apparent. Once the list of indicative uncertainties had been approved, suggested ‘uncertainties’ that could already be resolved by published systematic reviews were removed from the process. To assess which groups [patients, nurses, dietitians, doctors] were submitting uncertainties, data were collated on responder prevalence for particular uncertainties.

### 2.4. Stage 4. Ranking of treatment uncertainties

The aim of this stage was to rank the indicative uncertainties into those that both patients and health care professionals felt were important. The list of indicative uncertainties was converted into a survey, which was sent out to patients, nurses, gastroenterologists, surgeons and dietitians using the same mechanisms to contact participants engaged in stage 2. Participants were asked to select a maximum of 10 uncertainties that they considered to be the most important and to rank these from first to 10th in order of priority. Results of the ranking exercise were then reviewed by the steering group and an agreed priority list was established based on an overall rank order when ordered by frequency of votes. In the event of a large difference between the number of patients and health care professionals taking part, it was pre-agreed that the ranked lists would be examined both separately and combined for all participants. However, this did not prove necessary.

### 2.5. Stage 5. Development of list of ‘Top 10 research questions in IBD’

A final 1-day workshop was convened with the aim of developing a list of the ‘Top 10 Research Questions in IBD’. All steering group members were invited to participate in the workshop, in addition to partner organisations, including the National Institute for Health Research [NIHR]. Researchers from NIHR with experience in the design and conduct of applied clinical research assisted in the development of well-formulated and feasible research questions. Further invitations were made to ensure a balance of patients and clinicians. Participants were sent the list in advance so they could individually consider research questions before the workshop. Members of the group who were not patients or clinicians could take part in discussions but did not have voting rights.

Participants were divided into four discussion groups. Each group was led by an independent facilitator, who was briefed to ensure that all participants had an opportunity to express their opinion, and was made up of patients, clinicians and researchers. Each of the groups was asked to address and discuss several treatment uncertainties and rank them in order of priority. Led by a facilitator, the whole group then ranked the overall set of uncertainties. Cards with uncertainties written on them were laid out in rank order and moved around by the facilitator while the rank order was actively debated until consensus was reached regarding the uncertainties to be removed and the order of remaining priorities. Participants were asked to focus on agreeing a top 10. This was the final chance for participants to make a case for any particular uncertainty and its position in the top 10 before the final ranked list was produced.

## 3. Results

### 3.1. Collection of treatment uncertainties

A total of 1636 uncertainties were collected in the initial survey from 531 respondents. It was not mandatory to specify the profile [i.e. patient/ carer/ health care professional] of the respondent. Of the 519 respondents who did specify their profile, 113 were health care professionals or researchers and 406 were patients, carers or patient organisation representatives. Therefore, 22% of respondents were health care professionals and 78% were patients or carers. Of the health care professionals who specified their role, 6 specified themselves as dietitians, 3 as general practitioners, 13 as nurses, 13 as surgeons and 70 as gastroenterologists.

### 3.2. Collation of treatment uncertainties

Uncertainties from UK Database of Uncertainties about the Effects of Treatments, Research Recommendations from Cochrane Systematic Reviews, and the National Institute for Health and Care Excellence, which were not represented in the list of uncertainties from the survey were added, giving a total of 1671 uncertainties. Steering committee members removed 418 uncertainties which were ‘out of scope’, for example any non-questions [e.g. statements or comments], uncertainties not relating to IBD or uncertainties not related to treatment of IBD [e.g. aetiology, social or political questions]. The remaining 1253 uncertainties were classified according to the Health Research Classification Scheme, which is a system for classifying the biomedical and health research across all areas of health and disease. Many of the submitted remaining uncertainties were similar to others, therefore similar uncertainties were aggregated and collated by the independent information analyst to distil the key theme of each indicative final uncertainty. This penultimate longlist was then reviewed by two gastroenterologists to ensure fidelity to the submitted uncertainties, and sense was checked to be correctly framed, clinically and narratively. Following this stage, a total of 70 treatment uncertainties remained to go forward to the next round of the priority-setting process, the ranking survey and vote, followed by ranking based on weighted scores as outlined in stage 4 above. The top 25 topics were then put forward for the final workshop.

### 3.3. Development of list of ‘Top 10 research questions in IBD’: final workshop

A total of 16 people participated in the final workshop: three patients with IBD, two carers, three gastroenterologists, one colorectal surgeon, two IBD specialist nurses, two dietitians, one Crohn’s and Colitis UK charity representative, one information specialist and a James Lind Alliance facilitator. Additional observers who attended from the NIHR HTA group did not participate in the voting process but facilitated the participants, intervention, comparator, outcome [PICO]-setting process that followed.

### 3.4. Final results of priority setting: the top 10

1. What is the optimal treatment strategy considering efficacy, safety and cost-effectiveness [immunomodulators, biologics, surgery, combinations] in IBD management: selecting the right patient group, right stage of disease and assessing potential for withdrawal?
2. What are the optimal markers combinations of markers [clinical, endoscopic, imaging, genetics, other biomarkers] for stratification of patients with regard to: [a] disease course;[ b] monitoring disease activity; and [c] treatment response?
3. What role does diet have in the management of mildly active or inactive ulcerative colitis or Crohn’s disease to achieve normal daily activities and symptom control?
4. How can pain be most effectively managed in people with IBD?
5. What is an optimal treatment strategy for perianal Crohn’s disease and what individual factors determine this?
6. What is the best treatment for controlling diarrhoea and/or incontinence symptoms in people with IBD, including novel pharmacological and non-pharmacological options? Is high-dose loperamide safe and effective in the treatment of diarrhoea in IBD?
7. What is the optimal dietary therapy [liquid enteral diet and/or reintroduction diet] and duration to achieve mucosal healing in active IBD and/or remission, as either a primary or an adjunctive treatment? Is there a difference between adults and children?
8. What is the association between IBD and fatigue and how should it be managed?
9. Does early surgery or later surgery for terminal ileal Crohn’s disease result in better outcomes [quality of life, cost-effectiveness]?
10. Does influencing the gut microbiota influence the course of IBD?

For the first research question, two related questions were combined by consensus as there was a clear overarching theme: [a] are combinations of immunomodulators and biologics safe, and which will be most effective in IBD management? and [b] which immunomodulators or biologics are most effective in IBD management and what is the optimal treatment strategy: selecting/stratifying for the ‘right patient group’ at the ‘right stage of disease’/potential for withdrawal?

Similarly for the second research question, two related questions were combined by consensus as there was a clear overarching theme: [a] what are the optimal markers [clinical, endoscopic, biomarkers, genetics] that stratify patients with regard to disease course and treatment response? and [b] what are the optimal methods of monitoring disease activity in both active and quiescent IBD?

For the ninth research question, two related questions were combined by consensus as there was a clear overarching theme: [a] what are the relative roles of surgery and/or medication in the treatment of terminal ileal Crohn’s in terms of clinical and cost-effectiveness in quality of life, and what factors are important in decision making? and [b] is step-up or step-down treatment [bottom-up/bottom-down] more effective in IBD management and which is safer?

For the 10th research question, two related questions were combined by consensus as there was a clear overarching theme: [a] what is the role of antimicrobial approaches such as antibiotics in the management of IBD and is faecal transplantation effective in patients with IBD and how can it be optimized [delivery/donor]? and [b] are probiotics useful in the management of active or inactive IBD to achieve symptom control and normal daily activities?

Impact of diet on IBD was one over-arching topic which had particular discussion. It was highly rated by patients and clinicians as an area which needed more research. Additionally, there was an emphasis on addressing symptom control [pain, faecal incontinence and fatigue] by both patients and clinicians.

Of the research questions in the top 10, there was concordance between health care professionals and patients for most of the questions, including those related to diet, strategies of therapy [balancing benefit and risk] and role of microbial approaches. There were some examples of discordance between health care professionals and patients, with patients rating the questions as higher priorities than health care professionals. Such questions included those addressing symptom control, including incontinence and pain. Although health care professionals rated these questions highly, the ratings by patients enabled prioritisation within the top 10. This underlines the importance of having patients involved in the process of identifying research questions.

After the top 10 list was developed, with expertise from researchers from the National Institute for Health Research, some indicative research questions were designed according to PICO . The full list of these questions, formatted, is shown in Box 1. Clearly many more PICO questions can be developed based on these uncertainties.

**Box 1. List of 70 grouped uncertainties before final distillation.**

**Bacteria and IBD**

1. What is the role of antimicrobial approaches such as antibiotics in the management of inflammatory bowel disease? Is faecal transplantation effective in patients with inflammatory bowel disease and how can it be optimised [delivery/donor]?
2. Are probiotics useful in the management of active or inactive inflammatory bowel disease to achieve symptom control and normal daily activities?

**Constipation, diarrhoea and faecal incontinence**

3. What is the optimal treatment of proximal constipation in inflammatory bowel disease including effectiveness in controlling symptoms/quality of life?
4. What is the best treatment for controlling diarrhoea and/or incontinence symptoms in people with inflammatory bowel disease, including novel pharmacological and non-pharmacological options? Is high-dose loperamide safe and effective in the treatment of diarrhoea in inflammatory bowel disease?

**Diet and food**

5. What role does diet have in the management of active or inactive ulcerative colitis or Crohn’s disease to achieve normal daily activities and symptom control?
6. Is dietary therapy as effective as conventional treatment for maintaining remission in inflammatory bowel disease and what role does dietary modification have in symptom control?
7. What is the optimal dietary therapy [liquid enteral diet and/or reintroduction diet] and duration to achieve mucosal healing in active inflammatory bowel disease and/or maintain remission either as a primary or adjunctive treatment? Is there a difference between adults and children?

**Exercise and lifestyle**

8. Which lifestyle modifications, including exercise, have a role in maintaining remission in inflammatory bowel disease?
9. Which lifestyle modifications, including exercise, are safe and efficacious during disease relapse in inflammatory bowel disease, either as primary therapy or as an adjunct?

**Fatigue**

10. What is the association between inflammatory bowel disease and fatigue and how should it be managed?

**Information needs**

11. What are the information needs of people with inflammatory bowel disease and can education and support lead to improved clinical and quality of life outcomes?

**Medication uncertainties**

**Aminosalicylates [5-ASA drugs, e.g. mesalazine, olsalazine, balsalazide]**

12. What is the optimal strategy to improve adherence to aminosalicylates in ulcerative colitis?
13. Do aminosalicylates have a preventive effect against bowel cancer in Crohn’s disease?
14. Is low-dose aspirin safe and effective for prevention of inflammatory bowel disease-related colorectal cancer?
15. Are generic mesalazines less effective in treating inflammatory bowel disease than branded products?

**Immunomodulators and biologics**

16. Are combinations of immunomodulators and biologics safe, and which will be most effective in inflammatory bowel disease management?
17. Which immunomodulators or biologics are most effective in inflammatory bowel disease management? What is the optimal treatment strategy: selecting/stratifying for the ‘right patient group’ at the ‘right stage of disease’/potential for withdrawal]?

**Long-term safety of immunomodulators**

18. How safe is it to continue immunomodulators and biologic drugs in the long term?

**Low-dose naltrexone**

19. Is low-dose naltrexone safe and effective in the treatment of inflammatory bowel disease?

**Methotrexate**

20. Is methotrexate effective in the treatment of ulcerative colitis and how is it best administered: orally or by injection?
21. Are azathioprine and methotrexate equally effective in Crohn’s disease?

**Thiopurines [azathioprine and mercaptopurine] in ulcerative colitis**

22. What is the effectiveness of optimised thiopurine medications [azathioprine and mercaptopurine] in ulcerative colitis?

**Steroids**

23. What would be the strategy for use of new-generation steroids in the management of inflammatory bowel disease, weighing up benefits and risks?

**Medicine vs surgery**

24. What are the relative roles of surgery and/or medication in the treatment of terminal ileal Crohn’s disease in terms of clinical and cost-effectiveness in quality of life? What factors are important in decision making?

**Monitoring**

25. What are the optimal methods of monitoring disease activity in both active and quiescent inflammatory bowel disease?
26. What are the optimal markers [clinical, endoscopic, biomarkers, genetics] that stratify patients with regard to disease course and treatment response?
27. Does measuring metabolites and or adding allopurinol improve safety and efficacy of thiopurine therapy in inflammatory bowel disease?

**Other therapies**

28. Do complementary therapies, for example acupuncture, hypnotherapy or aloe vera, have a role in the management of inflammatory bowel disease to achieve symptom control and normal daily activities [active/remission/maintenance stages]?

**Pain**

29. How can pain be most effectively managed in people with inflammatory bowel disease?
30. Are there effective alternatives to non-steroidal anti-inflammatory drugs [e.g. ibuprofen] for the treatment of non-abdominal pain in patients with inflammatory bowel disease?

**Perianal Crohn’s disease**

31. What is an optimal treatment strategy for perianal Crohn’s disease and what individual factors determine this?

**Psychological support and therapy**

32. How do different psychological interventions compare in relation to improvements in quality of life, symptom control and clinical outcome markers in inflammatory bowel disease?
33. What is the evidence for the role of stress in flare-ups and how might this be managed?
34. Do counselling or support groups help patients with inflammatory bowel disease manage their symptoms and improve adherence?

**Reproductive health**

35. Does breastfeeding affect maternal remission maintenance?
36. What is the relationship between the menstrual cycle and inflammatory bowel disease flares?

**Self-management**

37. Is guided self-management effective and does it improve outcomes, quality of life and function in inflammatory bowel disease?
38. Do expert patients who look after themselves well, including diet and exercise, have a better outcome than those who simply take their medication as prescribed?

**Smoking and IBD**

39. Is there a therapeutic role for nicotine or cannabis in the treatment of inflammatory bowel disease?

**Stem cell treatment**

40. Is stem cell or gene therapy effective and safe in inflammatory bowel disease?

**Surgery**

41. Is laparoscopy more clinically effective and cost-effective than open surgery for small bowel Crohn’s disease, and how do the postoperative complication profiles differ?
42. Do all patients with active perianal Crohn’s disease need a seton before treatment with biologics, and if so, when should it be removed during the treatment course?
43. What are the consequences of colectomy in ulcerative colitis and how should they be managed?
44. What technical factors impact upon the timing and severity of postoperative recurrence of Crohn’s disease?
45. When is the optimal time to operate on patients with ileo-caecal Crohn’s disease?
46. How should ileo-anal pouch patients be stratified for follow-up?
47. How should perioperative medications [biologics, steroids, immunosuppressives] be optimised to improve outcomes and minimise complications?
48. What factors determine the decision to have a pouch post colectomy for ulcerative colitis?
49. Does ileorectal anastomosis have a place in the management of ulcerative colitis?
50. What is the optimal management of the rectal stump in subtotal colectomy?
51. Is segmental resection for Crohn’s colitis justified?
52. Is balloon dilatation or surgery best for small bowel strictures in Crohn’s disease?
53. Can appendectomy reduce the incidence of acute exacerbations in ulcerative colitis?
54. What is the optimum period to wait to operate after percutaneous draining of a Crohn’s intra-abdominal abscess?
55. What is impact of pouch surgery on fertility and sexual function?
56. How much macroscopic clearance is needed for Crohn’s resection to prevent early recurrences?
57. Does laparoscopic/laparoscopy-assisted restorative proctocolectomy have enough benefit over open surgery to be recommended as treatment of choice?

**Surveillance and cancer**

58. What is the best method of colonic surveillance in inflammatory bowel disease and what additional benefits do novel endoscopic technologies confer?
59. Which biomarkers have a role in colorectal cancer risk stratification?
60. Does good disease control alter cancer risk?
61. How effective are colonoscopic surveillance programmes in improving cancer related outcomes for patients with inflammatory bowel disease?

**Treatment after surgery**

62. How should we stratify patients for postoperative maintenance therapy after resection of small bowel Crohn’s disease?
63. What is the best way to manage short bowel symptoms caused by inflammatory bowel disease surgery?

**Treatment goals, outcome measures and delivery of care**

64. What are the best patient-reported outcome measures for the different groups of people with inflammatory bowel disease?
65. How do quality of life outcomes differ between competing strategies [medical or surgical]?
66. What are the optimal decision-making tools, for patients and clinicians in primary and secondary care, for inflammatory bowel disease treatment options?
67. Is step-up or step down-treatment [bottom-up/bottom-down] more effective in inflammatory bowel disease management and which is safer?

**Vaccines**

68. What should people with inflammatory bowel disease be vaccinated against?

**Supplements**

69. Are vitamin or mineral supplements effective in inducing or maintaining remission, for example vitamin D, in inflammatory bowel disease?

**Worms**

70. Is worm therapy safe and effective in inflammatory bowel disease, and in which patients?

## 4. Discussion

The paper outlines the top 10 research questions in the treatment of IBD which have been devised by clinicians and patients using the infrastructure and process of the James Lind Alliance Priority Setting Partnership. This exercise has never been previously applied to any gastrointestinal disease. IBD was selected as the lead topic as it is the most common serious chronic gastrointestinal [GI] disease and because the UK patient community is well organised and accessible via Crohn’s and Colitis UK and CICRA.

The top research priority, reached by consensus between patients and clinicians, reveals considerable concern in both groups about the relative efficacy and safety of commonly used IBD treatments, their appropriateness in different patient groups and their place in treatment pathways management. This is an uncertainty which frequently faces clinicians and patients, and this partnership confirms thatthese concerns are shared by each community. This evidence gap may reflect the low number of robust, industry-independent trials of treatment pathways in IBD, but also that efficacy within randomised controlled trials does not reliably predict effectiveness or patient satisfaction in the real world. The study highlights the need for more pragmatic trials that put widely used treatment pathways head-to-head. Such trials would yield important information for developing IBD management guidelines.

The second research priority addresses the importance of achieving predictors of disease course, activity and response to therapy. Treatment pathways and strategies are dependent on such knowledge. The area represents a particular unmet need in IBD research.

Some of the top 10 priorities included topics which may be perceived to be more ‘patient-centred’, such as effectively managing pain, faecal incontinence or fatigue: these were supported as priorities by clinicians and patients. The role of diet in IBD was also strongly supported by patients, as has been highlighted in previous research assessing the patient perspective.^9^ Clinicians and researchers recognise the need for clinical and patient-focused research, and the need to translate clinical advances into practical outcomes for patients, but often patients are not involved in the process of discussing and setting research priorities. The finding of such ‘patient-centred’ questions in the top 10 confirms findings from other priority-setting processes and demonstrates the importance of including those affected by the condition in research prioritisation exercises.

There were relatively few submissions concerning surgical management, although the perianal disease management uncertainty has a very surgical component. Surprisingly few submissions were received on complementary and alternative approaches to treating IBD, and these received no real support in prioritisation.

This research prioritisation process is strengthened by having respondents from various roles, ages and ethnic groups. The involvement of such a diverse mix of stakeholders, including all members involved in multidisciplinary teams in IBD, is viewed as philosophically correct and gives the resultant priorities legitimacy. However, there are limitations to the approach that should be considered. First, the process is by its nature selective. The response rate to the questionnaire cannot be determined, as the number of potential respondents reached online is not known. The questionnaire was internet-based, although paper versions were also available. The website was in English, which may have restricted access to respondents who were not able to read English, so this approach may exclude potential respondents from certain ethnic groups. Second, because of the large number of questions submitted, the approach was to merge very specific questions on related themes into broad questions that could be voted on. There are challenges in reducing the uncertainties down, and information may be lost in the process. Third, the process is time-consuming and relies on participants volunteering and committing their time to the process. Fourth, there may be a bias in the respondents. Finally, the survey was undertaken in the UK and there may be different research priorities in different countries with different health care systems. Indeed, different health care systems may impact on different priorities as perceived by both health care professionals and patients.

Following the priority-setting partnership in IBD, the next steps involve improving visibility of the top 10 IBD uncertainties and their researchable questions as regards potential funding bodies. The National Institute of Health Research has issued several commissioned calls for management of inflammatory bowel disease therapy based on the research priorities that have been documented in this work. A further challenge is to capture and disseminate, via the UK Database of Uncertainties about the Effects of Treatments [DUETs] and other mechanisms, the insightful and more detailed questions that have been lost as a result of merging. This unique data set is therefore open to the global IBD research community to consider and access in planning and executing clinical research.

## Funding

This work was supported by the British Society of Gastroenterology [BSG] and the UK inflammatory bowel disease charity, Crohn’s and Colitis UK, who provided core funding for the project in equal shares, with other members of the committee contributing time and expertise or facilities at no cost.

## Conflict of Interest

AH has acted as a consultant, advisory board member or speaker for AbbVie, Atlantic, Bristol-Myers Squibb, Celltrion, Falk, Ferring, Janssen, MSD, Napp Pharmaceuticals, Pfizer, Pharmacosmos, Shire and Takeda. She also serves on the Global Steering Committee for Genentech. AZ, ML, KK, AD and JS have no competing interests
